# Protective effects of the aqueous extract of Crocus sativus against ethylene glycol induced nephrolithiasis in rats

**DOI:** 10.17179/excli2014-510

**Published:** 2015-03-12

**Authors:** Bahareh Amin, Hanieh Moghri Feriz, Alireza Timcheh Hariri, Naser Tayyebi Meybodi, Hossein Hosseinzadeh

**Affiliations:** 1 Department of Pharmacology and Physiology, Faculty of Medicine, Sabzevar University of Medical Sciences, Sabzevar, Iran; 2 Pharmaceutical Research Center, School of Pharmacy, Mashhad University of Medical Sciences, Mashhad, Iran; 3 Medical Toxicology Research Center, School of Medicine, Mashhad University of Medical Sciences, Mashhad, Iran; 4 Department of Pathology, Research Center for Skin Diseases and Cutaneous Leishmanaisis, Emam Reza Hospital, Faculty of Medicine, Mashhad University of Medical Sciences, Mashhad, Iran; 5 Pharmaceutical Research Center, Department of Pharmacodynamics and Toxicology, School of Pharmacy, Mashhad University of Medical Sciences, Mashhad, Iran

**Keywords:** Crocus sativus, saffron, kidney stone, renal calculus, nephrolithiasis, hyperoxaluria, calcium oxalate, ethylene glycol, oxidative stress

## Abstract

This study evaluated the possible protective effect of *Crocus sativus* L. (saffron) in the treatment of renal calculi. Aqueous extract of saffron (25, 50 and 100 mg/kg, daily) was administered intraperitoneally in two regimens of protective or curative, using male Wistar rats. Urolithiasis was induced by ethylene glycol (% 0.75) in drinking water. Urine was collected for biochemical analysis and the kidneys were prepared for total lipid peroxide and histological evaluation. Ethylene glycol feeding resulted in an increased urine output, renal excretion of oxalate and decreased excretion of citrate and magnesium. Saffron did not show diuretic effect; however, it significantly reduced the elevated urinary oxalate in prophylactic (50 and 100 mg/kg) and curative (100 mg/kg) studies. Only the high dose of prophylactic regimen restored citrate concentration of urine. Increased number of calcium oxalate crystals deposits in the kidney tissue of calculogenic rats was significantly reverted by the prophylactic and high dose of curative saffron treatment. Malondialdehyde (MDA, a lipid peroxidation product) in the kidneys was increased following the lithogenic treatment; however, prophylactic (50, 100 mg/kg) and curative (100 mg/kg) regimens with saffron reduced the elevated levels of MDA. Results in the current study indicate that saffron can protect against ethylene glycol induced calcium oxalate (CaOx) nephrolithiasis. The mechanisms underlying this effect are mediated possibly through effect on the urinary concentration of stone-forming constituents and an antioxidant effect.

## Introduction

Kidney stone disease has become a rising problem and the third prevalent disorder affecting the urinary tract with high recurrence. This disorder involves a complex of events, such as crystal nucleation, supersaturation, growth, aggregation, retention within renal tubules and migration to the renal papillary surfaces (Moe, 2006[20]). The majority (up to 80%) of all stones are mainly composed of CaOx. Drawbacks of current treatments necessitate finding new drugs and many remedies, taken from plants have been reported to be effective treatments for urolithiasis with little adverse effects (Butterweck and Khan, 2009[4]).

Saffron, the dried stigma from the flower of *C. sativus *L*., *has been used as a famous dietary ingredient and coloring agent since ancient times (Abdullaev, 1993[1]). In folklore medicine, it has also recommended for numerous ailments including cough, asthma, menstruation problems, insomnia, pain, colic, chronic uterine hemorrhage, painful urination and kidney stone (Hosseinzadeh and Nassiri-Asl, 2013[9]). Avicenna, a Persian physician and the most famous philosopher-scientists of Islamic word, described details of a remedy, consisted of saffron plus honey prescribed to produce diuresis and facilitate passage of kidney stone (Mousavi and Bathaie, 2011[22]). The traditional uses of saffron are now supported by different studies. Saffron and its bioactive ingredients have been reported to possess anti-cancer (Abe and Saito, 2000[2]), anti-oxidant (Hosseinzadeh et al., 2009[12]), plasma lipid lowering effect and insulin resistance improvement (Sheng et al., 2008[28]), anti-convulsant (Hosseinzadeh et al., 2008[11]), anti-depressant (Hosseinzadeh et al., 2003[7]), memory improvement (Hosseinzadeh and Ziaei, 2006[15]), antinociceptive and anti-inflammatory properties (Hosseinzadeh and Younesi, 2002[14]; Hosseinzadeh and Shariaty, 2007[13]).

The present study evaluated for the possible therapeutic potential of aqueous extract of *C. sativus* in experimentally induced calcium oxalate urolithic in two regimens of prophylactic and curative in rats.

## Material and Methods

### Animals

Male Wistar rats weighing 250-300 g were obtained from the animal house of the School of Pharmacy, Mashhad University of Medical Sciences, Iran. Animals were kept in a 12 h light-dark cycle environment and were fed normal laboratory chow food *ad libitum*. All procedures were approved by Mashhad University of Medical Sciences and followed the Internationally Accepted Principles for Laboratory Animal Use and Care. 

### Materials

The stigmas of *C. sativus* L. was purchased from Novin Saffron Co. (Mashhad, Iran) and analyzed in accordance to ISO/TS 259-2. Hydrochlorothiazide (HCTZ) was a gift from Darupaksh Pharmaceutical Co., Tehran, Iran. Potassium citrate was purchased from Sepidaj Pharmaceutical Co., Tehran, Iran. Malondialdehyde was obtained from Fluca (Switzerland). Ethylene glycol (EG) and ammonium chloride (AC) were provided from Merck (Germany). All compounds were dissolved in normal saline (0.9%) immediately before injection. 

### Preparation of extract

Aqueous extract was prepared by mixing saffron powder with distilled water at a ratio of 1:50 respectively, shacked in an incubator for 48 h, centrifuged at a rate of 4000 g for 10min and the supernatant was separated. In this stage, sediment was suspended in the half amount of mentioned distilled water and process was repeated again. The later solution was added to first one and stored in a −20°C freezer. The total solution was freeze-dried for sublimating of solvent.

### Stone induction

Ethylene glycol plus ammonium chloride model was used to induce urolithiasis (Khan, 1997[17]).

### Experimental design

Sixty-six animals were randomly divided into eleven groups (I-XI) containing six rats each. Animals in group I received tap drinking water for 30 days (intact control). Animals in group II were treated with the high dose of aqueous extract for 30 days (saffron control). All remaining groups received calculi inducing treatment for 30 days, comprised of 0.75% v/v ethylene glycol and 1% w/v ammonium chloride in drinking water *ad libitum* for 3 days. Following this, treatment was switched to only 0.75% EG for 27 days. Stone forming control group (G III), as a negative control, received only EG plus AC. As a positive control, groups IV and V received potassium citrate (2.5 g/kg) and hydrochlorothiazide (10 mg/kg), respectively from 1^st^ day to 30^th^ day of calculi induction. Groups VI-VIII served as prophylactic treatment groups and received extract at doses of 25, 50 and 100 mg/kg respectively from 1^st^ day to 30^th^ day of calculi induction. Groups IX-XI were given aq. extract of saffron 25, 50 and 100 mg/kg respectively from 14^th^ day to 30^th ^day of calculi induction and served as curative treatment group. Extract and standard drugs were dissolved in distilled water and given once daily by intraperitoneal route. Dosage selection was based on previous studies (Hosseinzadeh and Younesi, 2002[14]; Hosseinzadeh and Shariaty, 2007[13]).

### Collection and analysis of urine for biochemical assays

All animals were kept in individual metabolic cages and 24 h urine samples were collected on 0, 1, 7, 14 and 30^th^ day of calculi induction treatment. The volume of urine, oxalate, citrate, phosphate, magnesium and protein excreted out, were measured at the respective time points.

Determination of diuretic activity was performed during the first 24 hours after administration of compounds and HCTZ (10 mg/kg) was used as a standard diuretic drug (Lipschitz et al., 1943[18]).

Following volume determination of urine, samples were stored at -20°C until analyzed.

### Collection and analysis of tissue for histological assays

After the last urine collection, animals were anaesthetized and killed. The kidneys were immediately excised and washed in ice-cold saline. The right kidneys were fixed in 10% neutral buffered formalin. The 5 μm thick sections were stained via a haematoxylin-eosin solution and examined by light microscopy. The instances of stone depositions were counted in ten microscopic fields and given a score as follows: Number of tubules filled by stone/total number of tubules in the field 100.

### Collection and analysis of tissue for measurement of lipid peroxidation

The left kidneys (200 mg) were worked into 10% homogenate in 1.15% potassium chloride solution (2 ml), to estimate lipid peroxidation by measuring the MDA content by thiobarbituric acid reactive method (Uchiyama and Mihara, 1978[32]). 3 ml phosphoric acid (1%) and 1 ml TBA (0.6%) were added to 0.5 ml of homogenate in a centrifuge tube. After mixing, all samples and standards (a set of MDA standards over the concentration range of 0-50 nmol/ml) were heated at 100°C for 45 min and cooled by using water. 4 ml n-butanol was added to the mixture and vortex-mixed for 1 min followed by centrifugation at 3000 rpm for 15 minutes. The organic layer was transferred to a fresh tube and its absorbance was read at 525 nm. Levels of MDA were expressed as nmol/mg of tissue.

### Statistical analysis

The results were expressed as mean ± standard error mean (SEM). The statistical significance was assessed using two-way analysis of variance (ANOVA) followed by Bonferroni's comparison test or by Student's *t*-test.The crystal deposit number and biochemical data were analyzed by one-way ANOVA followed by Tukey's for pair wise comparison. The value of P < 0.05 was considered significant. 

## Results

### Effect on the urine output

Before the start of treatment (day 0), biochemical parameters were not significantly different among all the groups. On day 1 after the stone induction, the reference diuretic drug, hydrochlorothiazide (10 mg/kg) induced diuresis as compared to intact control animals (P<0.05). Co-treatment with aqueous extract did not change urine output at this time indicating saffron could not have diuretic effect. 

Urinary volume was gradually increased after the stone induction treatment; however it was significantly higher than in the intact control group 30 days after administration (P < 0.05). Different dosages of aq. extract of saffron reduced urine volume of animals at this time although not to a significant extent (Table 1(Tab. 1)). 

### Effect on the urinary parameters

As indicated in Table 2(Tab. 2), 24-hour oxalate excretion was increased in negative control group and it was significantly higher than that in the intact control group 14 (P< 0.001) and 21 (P< 0.001) days after EG+AC treatment. Simultaneous treatment with 100 mg/kg of aqueous extract reduced the increase in urinary oxalate excretion on days 14 and 30 (P < 0.05, P < 0.001 respectively). Similar results were obtained when rats were treated with k-citrate as a positive control. Lower dose (50 mg/kg) in prophylactic regimen of saffron's extract reversed the increased levels of oxalate only on day 30 (P < 0.01). In curative regimen only the high dose of 100 mg/kg significantly (P < 0.01) reduced the elevation of renal oxalate content, on day 30 (Table 2(Tab. 2)). 

Increased excretion of phosphate in urolithic group was not statistically significant and remained comparable to that of intact control animals. Treated groups also did not show any statistically significant change in this parameter.

Urinary protein loss (proteinuria) was observed after 14 days of stone induction (P < 0.001) and remained significantly different until the 30^th^ day (P < 0.001), as compared to intact control group (Table 2(Tab. 2)). The co-administration of aqueous extract of saffron at the dose of 100 mg/kg in prophylactic study prevented the loss in protein on days 14 and 30 (P < 0.05) *vs*. lithogenic group. 

Urinary citrate excretion was gradually decreased by stone inducing treatment, as it was significantly lower than that of the intact control group 14 and 21 days after administration (P<0.05). However, supplementation with prophylactic regimen of 100 mg/kg of extract significantly prevented the decline in the levels of citrate on day 30 (P<0.05, Table 3(Tab. 3)). Potassium citrate, a well-known antilithiatic drug also normalized urinary citrate levels as compared to lithiatic control group (P<0.01).

Magnesium level of urine was not altered to a significant level in the lithiatic group and remained comparable to that of intact control animals (Table 3(Tab. 3)).

### Histopathologic findings

**Figure 1:**
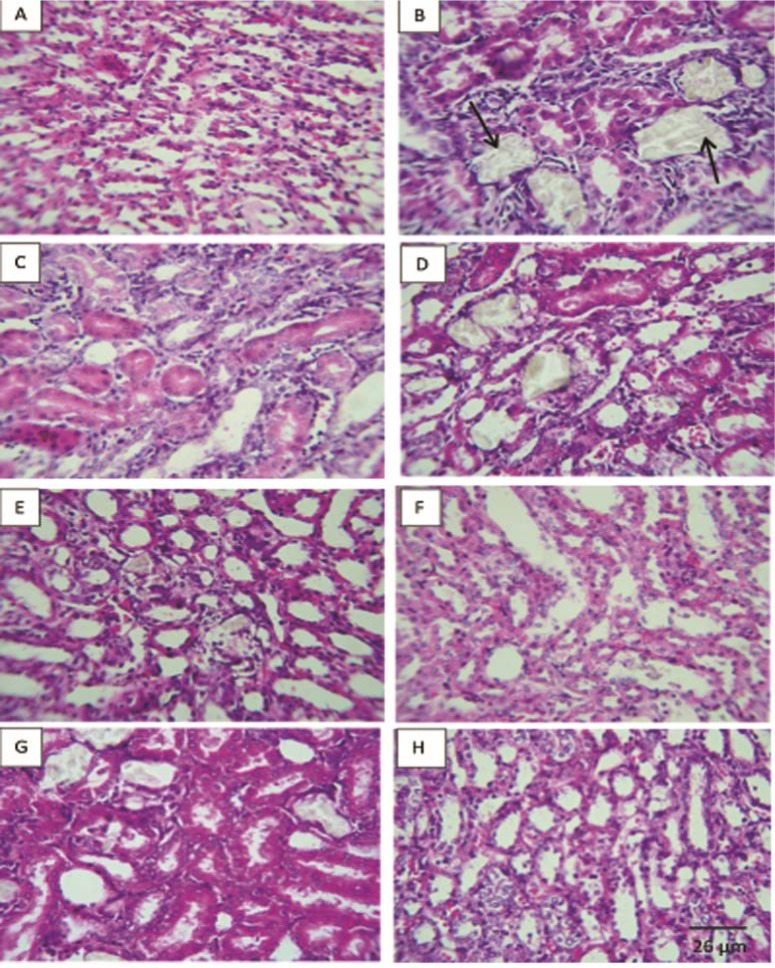
Represented light microscopy of paraffin sections viewed under polarized light of rat kidneys in each groups. Arrows indicate the crystals in the cortex, tubular, and medulla (x40) of rat at 30 days. There were no deposits in intact control group (A). Ethylene-glycol-induced urolithic group showed significant increased levels of crystal deposits (B). Positive control group (k citrate, C); Preventive treated groups of *Crocus sativus* aqueous extract (25, 50 and 100 mg/kg, D, E, F respectively) and curative treated groups (50 and 100 mg/kg, G, H respectively).

Examination of kidney paraffin sections showed that in the lithogenic treatment-induced group deposition of the calcium oxalate crystals in the renal tissue was increased with some interstitial infiltration whereas no crystal deposition was observed in intact control animals (Figure 1(Fig. 1) and Table 4(Tab. 4)).

However, prophylactic therapy with aqueous extract of saffron (50, 100 mg/kg) significantly (P < 0.01) and curative therapy partially dissolved the calcium oxalate stones in renal tissue (P < 0.05).

### Effect on the lipid peroxidation

**Figure 2:**
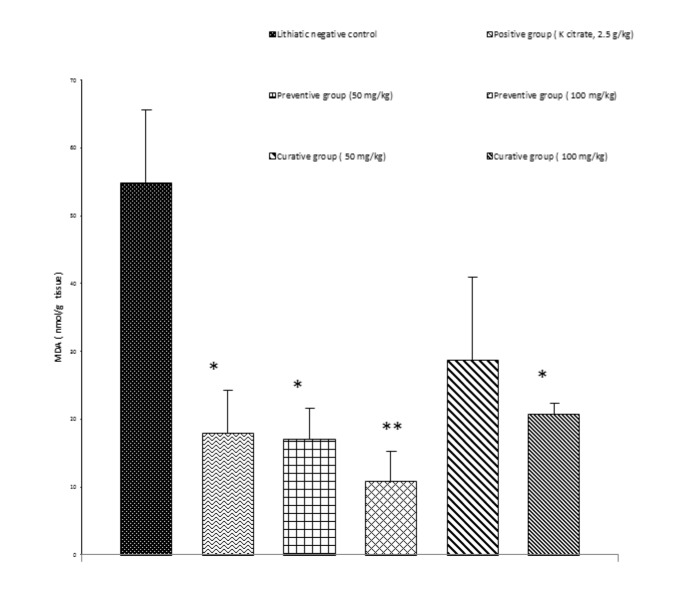
Concentration of MDA in the kidney tissue of different treated groups. Data was shown as mean ± S.E.M. Significance was determined by One-Way ANOVA followed by Tukey Post-hoc test: *P<0.05, ** P< 0.01 versus EG-treated group (n = 4).

Renal stone induction caused significant increase (P<0.01) in the lipid peroxidation of kidney tissue of the Group II as indicated with higher MDA levels (Figure 2(Fig. 2)). Animals receiving a simultaneous treatment with prophylactic (100 and 50 mg/kg, P<0.01, P<0.05, respectively) and curative regimens (100 mg/kg, P < 0.05) of extract had lower MDA levels as compared to lithiatic control.

## Discussion

The annual incidence and prevalence of nephrolithiasis is reported to be increasing worldwide, along with a decrease in the age of onset, perhaps due to the result of change in lifestyle, diet and climate (Zuckerman and Assimos, 2009[35]). Some natural therapeutic agents have been shown protective effects in different *in vitro* and *in vivo* models of urolithiasis possibly due to multiple constituents, acting through different pathways (Gandhi et al., 2013[6]; Hosseinzadeh et al., 2010[8]). As urinary system of male rats resembles that of humans, this genus was selected to induce urolithiasis (Khan, 1997[17]). Evidence in previous studies indicated that 14 days administration of EG, which increases urinary concentration of oxalate results into the formation of renal calculi composed mainly of calcium oxalate (Fan et al., 1999[5]; Khan, 1997[17]). The biochemical mechanism for this process is related to nonspecific dehydrogenase of EG, metabolized to oxalic acid and leads to hyperoxaluria, a major risk factor in the pathogenesis of kidney stones. It seems that hyperoxaluria provides an environment appropriate to stone formation by forming calcium oxalate deposition.

Ammonium chloride accelerating stone formation through metabolic acidosis, was added to the regimen of EG for 3 days (Fan et al., 1999[5]).

Oxalate excretion was progressively increased in EG/AC-treated groups. Whereas, on the administration of aqueous extract of saffron, oxalate excretion decreased in a dose dependent manner.

When tested for diuretic activity, HCTZ induced diuresis in rats over a period of 24 h. However, saffron did not increase urine output at this time. As in folklore medicine saffron has been said to possess diuretic activity, this effect might appear in higher or lower doses that were not evaluated in our study or after 30 days of the extract administration.

Urine level of citrate was lower in rats treated with EG/AC than intact control animals. Low level of citrate is an important risk factor for the stone formation in kidney (Zuckerman and Assimos, 2009[35]). Citrate complexes with calcium can increase its solubility and reduce free calcium content in the urine. Urinary citrate was improved by prophylactic treatment of high dose aqueous extract (100 mg/kg) similar to potassium citrate.

Urine elements such as phosphate and magnesium were not significantly affected by either urolithiasis or saffron extract treatments which is in agreement with some previous studies (Shirfule et al., 2013[29]).

The excretion of protein was increased in EG/AC treated animals and prevented by 30 days administration of aqueous extract of saffron (100 mg/kg). 

Microscopic examination of kidney sections derived from EG/AC induced urolithic rats showed crystal deposition in the kidneys of lithogenic group along with interstitial inflammation that might be attributed to oxalate. The concurrent administration of extract in prophylactic regimen (50, 100 mg/kg) prevented calcium oxalate crystal deposition in the kidney. The extract at a dose of 100mg/kg in the curative study also partially reduced the number of crystal deposition.

It has been indicated that high levels of oxalate and CaOx crystals lead to cellular injury, increased levels of reactive oxygen species and lipid peroxidation (Thamilselvan et al., 2003[30]). Lipid peroxidation has been suggested to be a predisposing factor for subsequent CaOx crystal deposition (Selvam and Bijikurien, 1991[27]). The exact mechanism(s) by which the saffron exerts its protective effects against EG-induced nephrolithiasis is not yet understood. In this study antioxidant potential of aqueous extract in the kidney of rats was evaluated by lipid peroxidation inhibitory activity. A significant increase in the MDA contents, representing lipid peroxidation, was observed in the kidneys of rats treated with ethylene glycol compared to that of intact control. Animals receiving aqueous extract of saffron exhibited lower MDA levels as compared to lithiatic control group. 

Although supplementation with the 50mg/kg of extract in prophylactic regimen and 100 mg/kg of curative regimen attenuated urinary oxalate levels and oxidative stress, none of them decreased proteinuria, probably due to the partial existence of kidney stones. Saffron active ingredients including crocetin, picocrocin (a glycosidic precursor of safranal), safranal, crocin (glycosides of the carotenoid crocetin) and other terpenoids, alkaloids and saponin contents may prevent the lipid peroxidation-induced renal damage caused by calcium oxalate crystal deposition in the kidney (Abe and Saito, 2000[2]; Alavizadeh and Hosseinzadeh, 2014[3]; Moraga et al., 2009[21]; Rezaee and Hosseinzadeh, 2013[26]). Antioxidant and anti-inflammatory activities of saffron and its bioactive components have been demonstrated in various studies. For example cerebral ischemia-induced oxidative damage was attenuated by safranal in the hippocampus of rats (Hosseinzadeh and Sadeghnia, 2005[10]). Cisplatin-induced nephrotoxicity prevented by 50mg/kg of the ethanolic extract of saffron and crocin through diminution of oxidative stress. Crocin prevented cytotoxicity induced by a potent neurotoxin, acrylamide, via decreasing apoptosis as well as ROS generation inhibition (Mehri et al., 2012[19]). In a study by Yoshino et al. (2011[34]), crocetin reduced reactive oxygen species in the brain of stroke-prone spontaneously hypertensive rats. Crocin and crocetin reduced LPS-induced nitric oxide (NO) release, TNF-α, IL-1β, intracellular ROS and NF-κB activation from cultured rat brain microglial cells (Nam et al., 2010[23]). Crocin showed antioxidant activity in the liver and kidney of streptozotocin-induced diabetic rats (Rajaei et al., 2013[24]). Thushara et al. (2013[31]) showed that platelet aggregation induced by oxidative stress was inhibited by crocin through an anti-apoptotic pathway. In another study, cyclophosphamide-induced organ toxicity was prevented by crocin through modulating antioxidant status and inflammatory cytokines (Jnaneshwari et al., 2013[16]).

Other potential protective effects of the extract might be due to the antispasmodic properties of flavonoids contents present in the saffron's extract, known to possess antispasmodic and Ca^2+^ channel blocking activities (Revuelta et al., 1997[25]).

Given that infection is probably to be associated with urolithiasis process, antimicrobial activity may also contribute to antilithiatic effect of saffron (Vahidi et al., 2010[33]).

Taken together it is suggested that aqueous extract of *Crocus sativus* could be useful as either alternative or an adjunctive therapy in the management of kidney stones. However, the exact mechanisms underlying this effect are still unknown, but are apparently related to antioxidant property and lowering of some urinary concentrations of stone forming constituents. 

## Acknowledgements

Authors are thankful to the Vice Chancellor of Research, Mashhad University of Medical Sciences for financial support. The results described in this paper are part of a Pharm. D. thesis.

## Conflict of interest

None declared.

## Figures and Tables

**Table 1:**
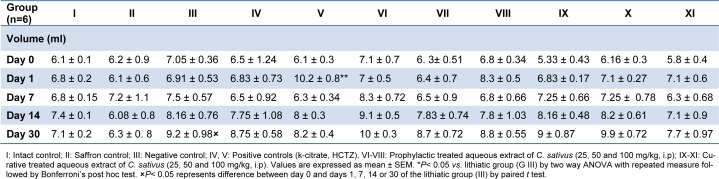
Effect of aqueous extract of *C. sativus* on volume (ml) of rats in the urolithiasis induced rats

**Table 2:**
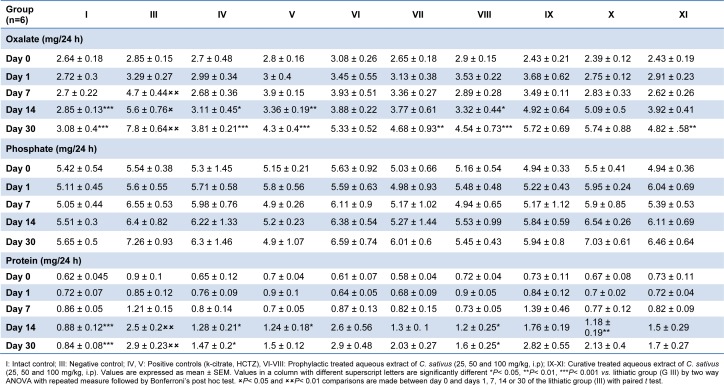
Effect of aqueous extract of *C. sativus* on urinary oxalate, phosphate and total protein levels in urolithiasis induced rats

**Table 3:**
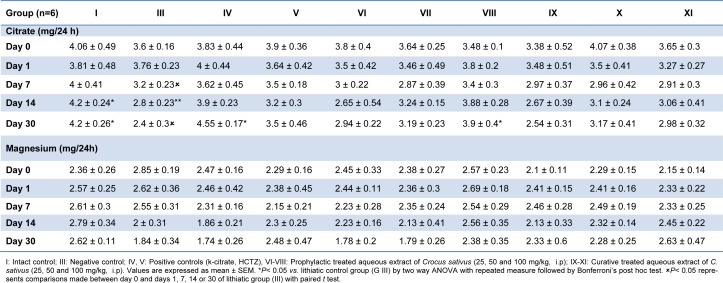
Effect of aqueous extract of *C. sativus* on urinary citrate and magnesium levels in urolithiasis induced rats

**Table 4:**
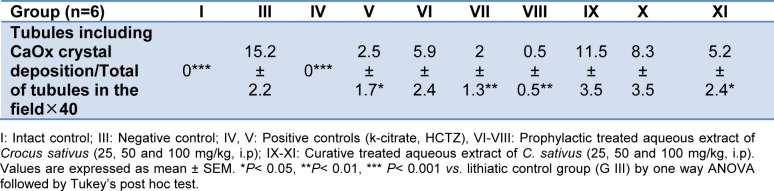
Effect of aqueous extract of *Crocus sativus* on the CaOx crystal deposition number in urolithiasis induced rats
